# Mind-Wandering and Its Relationship With Psychological Wellbeing and Obsessive-Compulsive Symptomatology in the Context of Covid-19

**DOI:** 10.1177/00332941231203563

**Published:** 2023-10-03

**Authors:** Niamh Gaynor, Lisa Fitzgerald

**Affiliations:** School of Psychology, 8818 Dublin City University, Dublin, Ireland

**Keywords:** Mind-wandering, obsessive-compulsive symptomatology, wellbeing, Covid-stress

## Abstract

Mind-wandering (MW) as a research topic has received considerable attention over the last several decades. The recent differentiation between spontaneous and deliberate MW has suggested a particular effect of the former on psychopathology; in that increased spontaneous MW may precede mental illness. The present study sought to explore MW as a potential contributing factor to poor mental health in the context of the Covid-19 pandemic. More specifically, we sought to determine firstly, whether the effects of MW frequency, type and content on subjective psychological wellbeing was consistent with previous findings after controlling for the impacts of Covid-related stress. Secondly, previous research has demonstrated an effect of both Covid-stress and spontaneous MW on the experience of obsessive-compulsive symptomatology (OCS), and so the present study explored this relationship further by assessing whether Covid-stress mediated the relationship between spontaneous MW and OCS. Participants completed measures of MW, OCS and psychological wellbeing through an online questionnaire. The results indicated that increased spontaneous MW was indicative of both poorer subjective psychological wellbeing and OCS, with Covid-stress partially mediating the relationship between spontaneous MW and OCS. Our findings provide further support for the adverse effect of unintentional MW on psychological wellbeing, as well as for the differentiation between both forms of the cognitive phenomenon. Additionally, they provide an important insight into one of the factors that may have preceded poor mental health among the Irish population during Covid-19. Future research may build upon the present study by exploring similar relationships among clinical populations.

## Introduction

### Stress and Wellbeing

In the 1940s, Hans Selye pioneered research on stress as a novel psychological concept impacting physiological health (Selye, 1956); laying the foundations for later researchers to establish a link between the cognitive phenomenon and psychological wellbeing (e.g., Mason et al., 1961). This relationship is now very well-established and frequently referred to in both academic and domestic circles; with stressors relating to one’s occupation (Babazono et al., 2005; Baumert et al., 2014; Lin et al., 2016), romantic relationships, interpersonal difficulties (Low et al., 2012) and socioeconomic status (Businelle et al., 2014) contributing to risk of suicide, depression, burnout and poor overall mental health among the general population. These examples reflect a mere fraction of the seemingly limitless number of stressors implicated in research as negatively impacting psychological wellbeing. However, a large proportion of them can be characterised as ‘environmental’ stressors; in that they each refer to one’s life experiences and the context in which they exist. Research exploring stress and wellbeing in the present-day cannot adequately do so without addressing the influence of contextual factors relating to the Covid-19 pandemic. In January of 2020, Covid-19; an acute respiratory disease caused by the novel coronavirus, became an international public health emergency (WHO, 2021), impacting the lives of the world’s population in an array of ways. Although the impact of the pandemic on mental health and wellbeing as a topic is in its relative infancy as of yet, there is already a wealth of research beginning to accumulate conveying the psychological burden associated with Covid-19. Members of the public have experienced relapses in depression, a decline in areas such as life satisfaction and positive affect, and increased risk and experience of depression, emotional disorder symptomatology, anxiety, stress, and psychological strain (Bendau et al., 2021; Fountoulakis et al., 2021; Gallagher et al., 2020; Patel et al., 2022; Planchuelo-Gomez et al., 2020; Shevlin et al., 2020; Zacher & Rudolph, 2020; see Robinson et al., 2022 for a review), each as a result of the circumstances surrounding COVID-19.

The term ‘Covid-related stress’ has been coined to describe the psychological and physiological aspects of stress that have been directly caused or exacerbated by the circumstances surrounding the Covid-19 pandemic. Internalisation of problems, increased pessimism (Asanjarani et al., 2021), psychological inflexibility (Arslan et al., 2021), negatively valenced dreams (Kennedy et al., 2021) and depressive symptoms (Lu et al., 2022) are just some of the issues associated with increased Covid-related stress as reported in emerging research. As outlined by Wheaton et al., (2012), the experience of psychological symptoms traditionally associated with stress and anxiety can prove adaptive in the face of widespread outbreak of contagious disease, in that it can encourage caution and engagement in health-promoting behaviour (e.g. Rubin et al., 2009). However, the distinction between this form of distress and an exaggerated psychological response is an important one to make, as excessive avoidance of potential contaminators and engagement in increased safety behaviours such as decontamination can exacerbate or trigger fear and health anxiety (Deacon & Maack, 2008; Gangemi et al., 2012; Olatunji et al., 2011). A potential consequence of increased health anxiety in the context of Covid-19 is the exhibition of symptoms similar to those of Obsessive Compulsive Disorder (OCD) among members of the general public. OCD is characterised by thoughts and compulsions that often centre around fears of contamination; a symptom that up to 56% of individuals with OCD will experience at some point (Mataix-Cols et al., 2002). The increasing exacerbation of obsessive, compulsive and anxiety symptoms has been observed among those with an existing clinical diagnosis of OCD as a result of the pandemic (e.g. Davide et al., 2020; French & Lyne, 2020; Wheaton et al., 2021), but there is also tentative evidence to suggest that Covid-related stress has, in some cases, manifested itself in non-clinical populations in the form of Obsessive-Compulsive symptomatology (OCS) (Abba-Aji et al., 2020; Cox & Olatunji, 2021; Cunning & Hodes, 2022; Munk et al., 2020; Wheaton et al., 2021; Zheng et al., 2020). Recent research has also demonstrated the effect of health anxiety on OCD in the context of the Covid-19 pandemic, with a study by Hassoula and colleagues (2022) finding that increased experience of health anxiety, and poorer perceived consequences of the pandemic, were each related to increased hand-washing behaviour and distress avoidance among individuals with clinically significant obsessive-compulsive symptoms.

The results obtained in studies exploring this relationship are relatively mixed, in that some have found a significant association between Covid-19 and OCD/OCS (e.g., Abba-Aji et al., 2020; Cox & Olatunji, 2021; see Cunning & Hodes, 2022 for a review), while others have not (e.g., Chakraborty & Karmakar, 2020). A potential explanation for this apparent conflict, as put forward by Chakraborty & Karmakar (2020), is the type of contamination fear an individual with OCD experiences. In other words, such an individual may present with obsessions that relate to dirt and germs for reasons not relating to fear of contracting a virus. Additionally, the latter study was entitled ‘Impact of Covid-19 on OCD’, yet they measured contamination fears and consequent compulsions alone, while studies by Abba-Aji et al., (2020) and Cox & Olatunji (2021) explored broader subtypes of OCD symptom categories (e.g., OCIR; hoarding, checking, ordering, neutralising, washing, obsessing). The aforementioned studies were also conducted in different countries (i.e., Canada vs. Iran), meaning that lockdown measures and communications from government officials may also have impacted incidence of Covid-related stress.

Discerning the extent to which Covid-related stress is truly responsible for declining mental health is challenging. Having participants report their experiences retrospectively, as some studies have done (e.g., Sauer et al., 2020), may not provide an accurate picture of their mental state due to issues such as subjective bias or inaccurate memory. Some studies (e.g. Mahamid & Bdier, 2021; Mancevska et al., 2020) have merely explored mental health across varying populations in the *context* of Covid-19 without including a measure related to the pandemic. Although results obtained from studies such as these provide a snapshot in time of how people may have been feeling while the pandemic was ongoing, it does not provide us with information regarding how Covid-19 has directly impacted mental health. Additionally, many studies have explored the experience of PTSD among varying populations, noting an increase in the phenomenon as a result of Covid-19 (e.g. Blekas et al., 2020; Cao et al., 2022; Di Crosta et al., 2020; Liu et al., 2020; Tang et al., 2020; see Grant et al., 2022 for a review). It could, however, be argued that the term ‘Post-Traumatic’ does not apply in this context due to the ongoing status of the pandemic in many parts of the world at the time of data collection. As such, a more effective means by which to explore its impact on varying aspects of psychological wellbeing may be to use measures that focus on Covid-related stress, anxiety or worry. Doing so ensures that participants are directly reporting their feelings and emotions surrounding Covid-19 in the moment, as opposed to attempting to report the extent to which they believe their mental state has changed over the last year. Additionally, the conflicting results and overall dearth of contextual research exploring potential contributing factors to OCS among the general population necessitates further investigation. Much of the empirical studies identified prior to data collection for the present study exploring this phenomenon did so in 2020, during the ‘first waves’ of the pandemic. Many countries had since experienced second, third and fourth waves, and new variants of the virus had been identified in several regions. Additionally, a significant proportion of the studies identified have focused on how Covid-19 has impacted those with existing diagnoses of OCD (e.g. Davide et al., 2020; French & Lyne, 2020; Khosravani et al., 2021; Liao et al., 2021). As such, more up-to-date, contextual research may be required in order to obtain a more comprehensive and relevant overview of the issue.

### Mind-Wandering

The role played by cognition, more specifically mind-wandering, in determining an individual’s experience of poor psychological wellbeing or OCS during Covid-19 is one that has not yet been examined to the best of our knowledge. Mind-Wandering (MW), often colloquially referred to as daydreaming, is a phenomenon in which an individual’s attention is diverted from the present to self-generated thoughts not associated with an individual’s immediate environment (Murray et al., 2020), and is believed to occupy up to 50% of our waking hours (Kane et al., 2007; Killingsworth & Gilbert, 2010). The shift in attention from the task at hand to stimulus-independent thoughts that occurs during MW has been widely cited among the literature as having adverse impacts on mental health and overall psychological wellbeing. Increased MW frequency has been associated with poorer wellbeing and negative affect across a range of studies (e.g. Deng et al., 2014; Killingsworth & Gilbert, 2010; Poerio et al., 2013), and may contribute to depressive (Deng et al., 2014; Seli et al., 2019) and dysphoric mood states (Smallwood et al., 2007). Additionally, daydreaming about those not close to us (i.e., fictional characters, romantic interests) may result in a heightened sense of loneliness and negative affect (Mar et al., 2012). Therapeutic practices that seek to treat depressive symptoms often emphasise mindfulness as an effective means by which to do so (see Hofmann et al., 2010 for a review), and it is therefore unsurprising that the antithesis of mindfulness, i.e. not living in the present and instead allowing your mind to wander to the past or the future, is associated with poorer mental health. However, it must also be noted that daydreaming about close friends and family can predict increased happiness, life satisfaction and feelings of connectedness, and limit feelings of loneliness (Mar et al., 2012; Poerio et al., 2016), and so it is possible that MW may relate to better psychological wellbeing in some contexts.

There is little information currently available on whether daydreaming frequency, type or content has influenced psychological wellbeing and mental health in the context of Covid-19. Preliminary research exploring MW as a distinct phenomenon has indicated an increase in the implementation of maladaptive daydreaming as a means by which to cope with the pandemic (Margherita et al., 2022; Musetti et al., 2021; Somer et al., 2020), but this appears to be the extent to which the construct has been directly explored. Boredom often acts as a precursor to mind-wandering (e.g., Jackson & Balota, 2012; McVay & Kane, 2009). As such, it is possible that the boredom potentially attributable to restrictive lockdown measures (e.g., Struk et al., 2020) has resulted in an increase in MW among the general population. Additionally, Covid-19 lockdowns may have exacerbated feelings of loneliness among the general public (Killgore et al., 2020). Periods of loneliness are often followed by an attempt to restore a sense of social connectedness, which may sometimes be done by daydreaming about those close to us (Poerio et al., 2016). It is therefore possible that the loneliness experienced by the world’s population during periods of lockdown and isolation has resulted in an increase in engagement in MW, as well as MW that is centred around our loved ones; emphasising the need to explore how this may have impacted psychological wellbeing in the context of the current pandemic.

MW has been primarily explored as a unitary cognitive phenomenon encompassing frequency alone, which may provide a basis on which we can begin to understand why daydreaming has so often been associated with poor mental health and negative affect. This failure to dissociate varying facets of MW from each other (i.e. type or content) has resulted in an abundance of research and papers portraying engagement in MW as posing no benefit, yet an increase in the research of the phenomenon in more recent years has provided results to the contrary (e.g. Smallwood & Andrews-Hanna, 2013). More recent research has also elucidated the potentially bidirectional nature of the relationship existing between MW and wellbeing, in that negative mood states may act as both a precursor to, and a product of MW (e.g. Banks et al., 2016; Kruger et al., 2020; Poerio et al., 2013; Smallwood & Schooler, 2006). Further investigation into the varying facets of MW has also highlighted the complexity of its measurement. MW has often been referred to as a deficit in attentional or executive control (e.g., McVay & Kane, 2009), yet this classification fails to account for those who *choose* to allow their minds to wander, and instead classes all MW as the failure of an individual to control their on-task behaviour. In the case of spontaneous MW; the type of MW in which an individual’s attention is unintentionally diverted from the present, this description may apply, but the recent differentiation between spontaneous and deliberate MW highlights the conscious choice made by some individuals to engage in the practice (see Seli et al., 2016 for a review). This distinction is supported by a wealth of research that shows the variation in effects of both types of MW on wellbeing outcomes. Spontaneous MW is more frequently associated with poor mental health than deliberate MW, with studies highlighting the positive association between the former and depression, anxiety, ADHD and OCD (Seli et al., 2015a, 2019). Additionally, those who engage in spontaneous MW more frequently than deliberate MW report increased experience of stress (Seli et al., 2019); a finding that may bear particular relevance to the Covid-19 context. Based on the above outlined evidence, it is possible that the intentionality of mind-wandering episodes may serve to explain the negative association of MW with affect and wellbeing. Its relationship with stress may also prove transferable to the present context, in that increased engagement in spontaneous MW may have resulted in increased Covid-related stress, and thus the experience of OCS.

### The Present Study

To date, no empirical study exploring the relationship between MW and wellbeing variables in the context of the Covid-19 pandemic has been conducted. We know from previous research that the Covid-19 pandemic has had a negative impact on the wellbeing of the world, impacting both subjective psychological wellbeing and psychopathology in the form of depression, anxiety and OCD. The link between varying facets of MW and wellbeing outcomes is also well-established, and its effect on stress may be transferable to the present context. Exploring both phenomena (i.e. MW and Covid-related stress) within the same context may provide a deeper insight into the relationship between MW and wellbeing outcomes during the pandemic, as well as determining how Covid-related stress may have influenced this relationship. The overarching research question we seek to answer is whether Covid-related stress has impacted the relationship between varying facets of MW (i.e. frequency, type and content) and psychological wellbeing, defined both by subjective psychological wellbeing and by the experience of obsessive compulsive symptomatology. Research has indicated that spontaneous MW and Covid-19 have each individually influenced OCS among clinical and non-clinical populations, but little attention has been paid thus far to the mechanisms underlying this relationship. Additionally, given that Covid-related stress has demonstrated a negative effect on both OCS (e.g. Khosravani et al., 2021) and wellbeing (e.g. Coiro et al., 2021), and that spontaneous MW is closely linked with wellbeing (Seli et al., 2015a, 2019), stress (Seli et al., 2019) and OCS outcomes (e.g. Abba-Aji et al., 2020), it is plausible that in the present context, Covid-related stress may mediate the relationship between spontaneous MW and OCS among our sample. We therefore seek to ascertain whether Covid-related stress has influenced the relationship between spontaneous MW and OCS, expecting that spontaneous MW will indirectly effect OCS through increased Covid-related stress. We also seek to determine whether daydreaming content has influenced wellbeing in the context of Covid-19. Daydreaming about close family and friends has been shown to be positively associated with life satisfaction (Mar et al., 2012), connectedness (Poerio et al., 2016) and happiness (Poerio et al., 2015), and so we hope to establish whether daydreaming about loved ones also impacts subjective psychological wellbeing, while simultaneously controlling for the possible effects of Covid-related stress. We expect to find that daydreaming about those close to us will be indicative of higher wellbeing. Finally, we wish to further explore the relationship between daydreaming frequency and type with wellbeing, as well as exploring whether this relationship remains consistent with previous findings even when Covid stress is controlled for. We anticipate that increased daydreaming frequency and intentional mind-wandering will each be negatively associated with wellbeing, after controlling for the effects of Covid-related stress.

## Methods

### Design

A cross-sectional survey design has been employed in the present study, as we sought to investigate the extent to which varying MW styles can account for or influence psychological wellbeing in the context of the Covid-19 pandemic. The independent variables included Covid-related stress, MW frequency, style (i.e. deliberate or spontaneous) and content (i.e. daydreaming about close family and friends); while the dependent variables included subjective psychological wellbeing and OCS. Information pertaining to gender, age and perceived social support was also collected.

### Participants

A random sampling method of recruitment was employed for the purpose of the present study, in that participants were recruited primarily online through the student researcher’s social media channels (i.e. Twitter, Facebook and LinkedIn). Advertisements were also posted on course notice boards, accessible only to Undergraduate and Postgraduate psychology students attending Dublin City University. Advertisements outlined a brief synopsis of what the study sought to explore as well as inclusion criteria, and included a link to the survey that individuals could access were they interested in participation. Participants were deemed eligible for participation if they were living in Ireland, over the age of 18 and did not have a current diagnosis of a mental illness, as this may have adversely impacted the reliability, and generalisability of the results. A final sample of 177 participants fully completed all measures; a sample size that meets the recommended minimum threshold of 82 for research with four independent variables as outlined by Tabachnick and Fidell (2013, p. 123).

### Measures

#### Mind-Wandering

##### Mind-Wandering Frequency

Daydreaming frequency was measured using Singer & Antrobus’s (1970) Daydreaming Frequency Scale; a 12-item scale included among the 28 distinct subscales of the Imaginal Processes Inventory. Participants are required to respond to statements relating to daydreaming frequency (e.g. ‘I recall or think over my daydreams), noting the extent to which they engage in the practice on a 5-point Likert scale (e.g. ‘Infrequently’, ‘once a week’, ‘once a day’ etc.). Higher scores on the scale are indicative of increased daydreaming frequency. The scale has demonstrated good internal consistency and test-retest reliability (Giambra, 1993).

##### Mind-Wandering Type

Seli and colleagues’ (2015b) Mind-Wandering: Deliberate (MW-D) and Mind-Wandering: Spontaneous (MW-S) scales, which have both demonstrated good reliability (Seli et al., 2015b), provided a means by which to assess individual differences in daydreaming type in the present study. The 4-item measures have participants select which number on a scale of one to seven most accurately reflects their mind-wandering experience. Items included on the MW-D scale relate to intentional daydreaming, e.g. ‘I allow myself to get absorbed in pleasant fantasy’, while the MW-S scale measures unintentional daydreaming by having participants respond to statements such as ‘It feels like I don’t have control over when my mind wanders’, both using a 7-point Likert scale. Total scores are generated for each scale by adding up scores on individual questions, and higher scores on either scale are indicative of increased engagement in either type of mind-wandering.

##### Mind-Wandering Content

Social daydreaming content was assessed using subscales developed by Mar et al. (2012), in which participants are asked to note their response to statements such as ‘My internal thoughts involve my present romantic partner’ on a 7-point Likert scale. Subscales include daydreams centring around romantic partners, those close to us we see often, those close to us we see rarely, fictional characters, people and increased daydreaming when lonely. Scores were generated for each subscale using the method outlined by Mar and colleagues, in that a mean score was calculated to provide an overall score for each subscale. The primary scales used for the purpose of the present study were those measuring daydreams about close family and friends we see both often and rarely. In order to generate one single score representing overall daydreams about loved ones, a mean score was calculated by adding up scores on both of the above subscales and dividing by two. Higher scores are reflective of increased daydreaming about close family and friends.

#### Perceived Social Support

The International Support Evaluation List (Cohen et al., 1985) was used to measure perceived social support, doing so by having participants respond to statements such as ‘When I feel lonely, there are several people I can talk to’ using a 4-point scale with answers ranging from 0 (‘Definitely false’) to 3 (‘Definitely true’). An overall score was generated by adding up scores on each individual question, with half of the total number of questions being reverse-coded. Higher scores are indicative of increased perceived social support.

#### Covid-Related Stress

The Covid Stress Scale (Taylor et al., 2020); a scale that has demonstrated both reliability and validity in measuring the impact Covid-19 has had on stress (Taylor et al., 2020), was used to measure Covid-related stress in the present study. The measure comprises six distinct subscales, each assessing different domains of Covid-related stress; danger, socio-economic consequences, xenophobia, contamination, traumatic stress and compulsive checking. Scores from each subscale are added to generate one overall scale score, and higher scores reflect increased Covid-related stress.

#### Psychological Wellbeing

##### Obsessive Compulsive Symptomatology

The Dimensional Obsessive Compulsive Scale (Abramowitz et al., 2010) was used to assess the experience of obsessive-compulsive symptoms among participants in the present study. The scale has demonstrated efficacy in measuring the above among both clinical and non-clinical populations in online environments (Enander et al., 2012), and is said to employ a more multidimensional approach to the measurement of symptom severity when compared to other commonly used measures such as the OCI-R (Abramowitz et al., 2010; Foa et al., 2002). The scale comprises a 20-item self-report measure with four distinct subscales measuring concerns about germs or contamination; being responsible for harm, injury or bad luck; symmetry, completeness and need for things to be ‘just right’; and unacceptable thoughts. Each subscale contains five items that are scored from 0 to 4. Scores are generated by adding up total scores for each subscale, resulting in one overall score ranging between 0 and 80. A cut-off score of 18 distinguishes between those exhibiting clinically significant OCD symptoms and non-clinical adults, accurately classifying 78% of both OCD patients and non-clinical adults.

##### Subjective Psychological Wellbeing

Finally, the 14-item Warwick Edinburgh Mental Wellbeing Scale (WEMWBS; Tennant et al., 2007) was used to measure subjective psychological wellbeing. Tennant and colleagues operationally define mental wellbeing as a construct that encompasses ‘autonomy, self-acceptance, personal growth, purpose in life and self-esteem’ as opposed to the absence of mental illness alone. As such, the term ‘wellbeing’ in the present study represents both the above outlined factors, as well as scores on the WEMWBS. The measure has demonstrated both validity and reliability across a diverse range of populations and research questions (Stewart-Brown et al., 2011), and assesses wellbeing by having participants respond to statements such as ‘I’ve been feeling relaxed’ on a 5-point Likert scale ranging from ‘None of the time’ to ‘All of the time’. An overall total is generated by adding together scores on each individual question. As the WEMWBS is not used as a diagnostic tool, there are no cut-off scores distinguishing between those with good mental health and those with poor mental health. Higher scores, however, are reflective of better overall psychological wellbeing.

### Procedure

Recruitment for the present study primarily took place during the months of February and March, while Ireland was experiencing its third wave of Covid-19, and Level 5 restrictions limiting social interaction, the opening of non-essential retail, hospitality and educational institutions were in place. A link to the Qualtrics (www.qualtrics.com) study questionnaire was widely distributed across varying social media platforms (namely Twitter, Facebook, Instagram and LinkedIn) over a sampling period of roughly 2 months. Measures were entirely reliant on self-report, as participants completed the questionnaire in their own time using their own devices. Upon clicking on the link, participants were presented with a plain language statement (see Appendix B) and a consent form (see Appendix C). After informed consent was received, they were required to disclose their age and gender, before completing a number of questionnaires pertaining to their psychological wellbeing and daydreaming habits. A debriefing form (see Appendix E) containing information about the study and contact details for both the research team and for counselling services they may wish to access was presented to participants upon completion of the entire questionnaire. Failure to fully complete the questionnaire was deemed indicative of withdrawal of consent to participate, and therefore partially completed questionnaires were deleted before the data was exported to a third party software for analysis.

### Ethical Issues

Ethical approval for the present study was obtained on the 11th February 2021 from the Psychology Ethics Committee (PEC) in Dublin City University (please see appendix 1 for a copy of the letter confirming ethical approval). The primary ethical concern related to the present study was the personal and potentially triggering nature of some of the questions participants were required to respond to. This risk was managed by including the diagnosis of a mental illness as a criterion for exclusion, and by imploring those who may be unduly impacted by responding to questions about their mental health to refrain from participating in the study in the Plain Language Statement. Additionally, contact information for DCU counselling, Samaritans, the DCU PEC chairperson and for Text 50808 was provided at the end of the debriefing form should participants have felt as though they required further support upon completion of the study. Participant’s right to withdraw consent from participation at any point was also clearly communicated.

### Data Analysis

Data was initially cleaned and screened for missing cases prior to the conduction of statistical analyses. There were a total of 16 missing responses out of a total 354 (5%) in the MW-D scale; consisting of an equal 8 missing cases across the questions ‘I enjoy mind wandering’ and ‘I allow myself to get absorbed in pleasant fantasy’. These missing responses occurred due to the omission of a ‘forced response’ requirement for progression in the questionnaire on Qualtrics, resulting in the failure of several participants to respond to both questions. The most appropriate means by which to address this as recommended by Tabachnick & Fidell (2013) was multiple imputation, as data was missing not at random (MNAR). Five imputed datasets were created and analysed using SPSS (Version 27) software and mice 3.0 default settings (Van Buuren & Groothuis-Oudshoorn, 2011). Repeated estimates have been pooled into final estimates, and as results obtained from the original dataset and from pooled estimates are similar, results obtained from pooled data are reported.

Preliminary analysis was carried out to determine whether the data met each of the required assumptions for regression. Skewness and Kurtosis values were used to determine the assumption of normality, and were within an acceptable range (i.e. skewness <2 and kurtosis <4; Kline, 1998), while Pearson correlation analysis confirmed that study variables were not highly correlated with each other. All other assumptions of linearity, homoscedasticity, independence of residuals, and absence of multicollinearity and outliers were met, and so the data was deemed suitable for hierarchical multiple regression.

To explore the impact of MW frequency, type and content on subjective psychological wellbeing in the context of Covid-19, a hierarchical multiple regression was conducted using IBM SPSS (Version 27) Statistics software. Covid stress was entered at Step one so as to control for its effects, daydreaming frequency at step two, spontaneous MW at step three, and daydreaming about close friends and family in the fourth, final step. The variables were entered in this order as daydreaming frequency is the most basic means by which to explore the construct, and has frequently demonstrated a negative relationship with wellbeing, whereas the relationship between daydreaming type or content and wellbeing is considerably less established. Spontaneous MW has demonstrated a negative effect on other wellbeing outcomes (e.g. stress, OCD, depression and anxiety), while daydreaming about close friends and family appears only to have been explored in the context of loneliness and life satisfaction; providing justification for placing spontaneous MW in step three of the regression and daydreaming about close friends and family in the final step. Scores on the Warwick Edinburgh Mental Wellbeing Scale represented the wellbeing outcome variable.

To assess the relationship between MW type and OCS, Pearson product-moment correlations were first calculated to determine whether deliberate and spontaneous MW related to OCS. Deliberate MW demonstrated a non-significant relationship with OCS, and so spontaneous MW was included in the simple mediation model. The mediating effect of Covid stress was investigated using the PROCESS Macro (Model 4) for SPSS Version 27 (Hayes, 2018). The indirect effect of spontaneous MW on OCS was tested using a non-parametric bootstrapping method. Bootstrapping refers to a method of statistical analysis wherein a population parameter is estimated through repetitive random sampling with replacement from the dataset. When employing this method of analysis, the dataset is treated as though it were representative of the true population, and seeks to replicate potential scores within the true population with each random sample. For the purpose of the present study, 5000 re-samples were taken from the data to establish 95% confidence intervals (Hayes, 2018; Preacher & Hayes, 2008). Employing this method precludes the need for data to meet usual assumptions of normality (Hayes, 2018, p. 70), and so the data was deemed suitable for this form of analysis. Results of the mediation model were interpreted using standardized path estimates and squared-multiple correlations (*r*^2^), whereby .14 indicates a small effect, .39 a medium effect and .59 a large effect (Cohen, 1988). An alpha level of .05 was used for all statistical analyses.

## Results

11 participants did not fully complete each of the measures included in the survey, and so their data was deleted and excluded from further analysis. As a result, data from total of 177 participants was included (136 female, 40 male and 1 non-binary), with ages ranging from 18 to 78 (*M* = 34.42, *SD* = 14.25). All participants were living in Ireland at the time of recruitment. Mean scores for each of the included measures are depicted in Table 1 below.

**Table 1.** Descriptive Statistics for Included Measures.

	Mean	Std. Deviation	Minimum	Maximum
MW-D	17.46	5.84	4	28
MW-S	16.12	6.23	4	28
DDFS	35.99	11.25	12	59
CSS	30.82	10.02	0	103
DOCS	15.62	12.24	0	77
WEMWBS	43.90	9.77	15	70
ISEL	4.35	4.51	12	120

Note. MW-D = Mind-Wandering Deliberate Scale, MW-S = Mind-Wandering Spontaneous Scale, DDFS = Daydreaming Frequency Scale, CSS = Covid Stress Scale, DOCS = Dimensional Obsessive Compulsive Scale, WEMWBS = Warwick Edinburgh Mental Wellbeing Scale, ISEL = Interpersonal Support Evaluation List.

### Hierarchical Regression Exploring the Effect of Mind-Wandering on Psychological Wellbeing

A four-step hierarchical multiple regression was used to assess the ability of spontaneous MW, daydreaming about close family and friends, and MW frequency to predict wellbeing after controlling for the influence of Covid-related stress (see Table 2 below). Preliminary analysis indicated that assumptions of normality, linearity, multicollinearity, homoscedasticity, univariate outliers, multivariate outliers and independence of residuals were not violated. It was hypothesised that increased scores on daydreaming frequency and spontaneous mind-wandering measures would predict lower wellbeing, while daydreaming about close family and friends would predict higher wellbeing. A hierarchical multiple regression revealed that Covid-related stress accounted for 7.1% of the variance in wellbeing among our sample, *F* (1, 175) = 13.33, *p* < .001. The addition of Daydreaming frequency in step two contributed a further 5.1%, with an *R*^*2*^ indicating significance, *F* (2, 174) = 12.09, *p* < .001. Introducing spontaneous mind-wandering scores in step three explained a further 1.9% of variation in wellbeing scores, *F* (3, 173) = 9.50, *p* = .05, with a significant *R*^*2*^ value. Finally, after entering ‘daydreaming about close friends and family’ scores into the fourth step of the model, the model as a whole accounted for 13.3% of variance in wellbeing, *F* (4, 172) = 7.78, *p* < .001. However, this change in *R*^*2*^ was not significant (*p* = .126). In the final model, only Covid stress and spontaneous mind-wandering uniquely predicted wellbeing, with Covid stress indicating a higher semipartial correlation value (*sr* = −.22, *p* = .003) than spontaneous mind-wandering (*sr* = −.15, *p* = .034). Neither daydreaming about close friends and family nor daydreaming frequency uniquely predicted scores on the wellbeing measures. These results suggest that after controlling for the effects of Covid-related stress, as spontaneous mind-wandering increases, subjective psychological wellbeing decreases. Additionally, when taken collectively, daydreaming about close friends and family, mind-wandering spontaneously and daydreaming increasingly frequently may predict lower wellbeing. However, daydreaming about close friends and family does not significantly contribute to the ability of the model to predict wellbeing, and so similar effects may still be observed if this variable was removed (see Table 2 below).

**Table 2.** Hierarchical Multiple Regression Analysis Summary for Variables Predicting Wellbeing.

	*B*	*SE B*	β	*sr*	*R* ^*2*^ *Change*	*R* ^*2*^
Step 1					.07***	.07***
Covid stress	−.14	.04	−.27	−.27		
Step 2					.05**	.12**
Covid stress	−.12	.04	−.22	−.22		
Daydreaming frequency	−.20	.06	−.23	−.23		
Step 3					.02*	.14*
Covid stress	−.11	.04	−.21	−.21		
Daydreaming frequency	−.09	.08	−.10	−.08		
Spontaneous MW	−.30	.15	−.19	−.14		
Step 4					.01	.15
Covid stress	−.11	.04	−.22			
Daydreaming frequency	−.11	.08	−.12			
Spontaneous MW	−.32	.15	−.21			
Daydream content	1.06	.69	.11			

Note. ***p < .001, **p < .01, *p < .05.

### The Mediating Effect of Covid-Related Stress on the Relationship Between Spontaneous Mind-Wandering and Obsessive Compulsive Symptomatology

**Figure 1.**
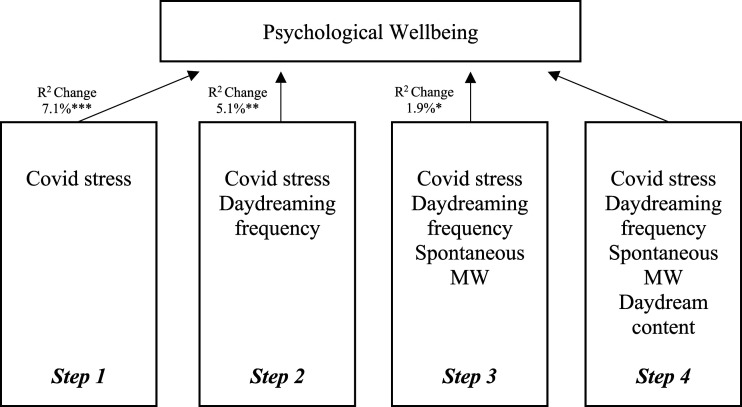
Hierarchical Multiple Regression Model depicting the effects of mind-wandering on psychological wellbeing after controlling for the effect of Covid stress. Note. ***p < .001, **p < .01, *p < .05.

Preliminary analysis exploring the association between mind-wandering type and OCS was done using a Pearson product-moment correlation coefficient. This analysis revealed no significant correlation between deliberate mind-wandering and scores on the DOCS (*r* = .08, *p* = .282), while spontaneous mind-wandering demonstrated a statistically significant positive association with OCS (*r* = .32, *p* < .001). Therefore, deliberate mind-wandering was not included in the subsequent mediation effect analyses. As depicted in Figure 1 below, results from a follow-up exploratory simple mediation model indicated that, as expected, spontaneous mind-wandering indirectly influenced obsessive compulsive symptomatology through its effect on increased experience of Covid-related stress, *indirect effect* = .22, *BSE* = .09, *p* = .005, 95% BCI [.05, .39]. Spontaneous mind-wandering positively predicted Covid stress (path a); *b* = .59, *t* (175) = 2.61, *p* = .0097, 95% CI [.15, 1.05], explaining 3.8% of the variance in Covid stress scores, *F* (1, 175) = 6.83, *p* = .0097, *R*^*2*^ = .038. Covid stress, in turn, positively related to OCD symptomatology (path b); *b* = .37, *t* (174) = 9.77, *p* < .001, 95% CI [.03, .04], accounting for 42.06% of the variance in OCD scores. The direct effect of Covid stress on OCD symptomatology (path c’) was statistically significant, *b* = .41, *t* (174) = 3.56, *p* = .005, as was the total effect of spontaneous mind-wandering on OCD (path c), *b* = .63, *t* (175) = 4.48, *p* < .001, accounting for 10.3% of the variance in OCD scores (see Figure 1 below).

**Figure 2.**
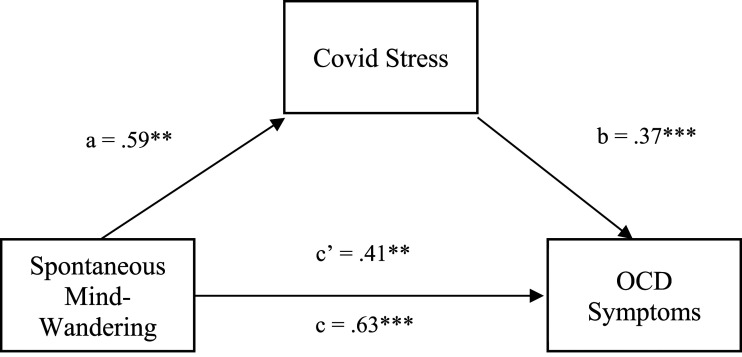
Simple Mediation Model for the Relationship Between Spontaneous Mind-Wandering and OCD Symptomatology as Mediated by Covid Stress. Note. ***p < .001, **p < .01, *p < .05.

The addition of Covid stress as a mediating variable considerably diminished the magnitude of the direct effect of spontaneous mind-wandering on OCD symptomatology (.63 > .41). As such, the results of the simple mediation model are consistent with our hypothesis that Covid-related stress partially mediates the relationship between spontaneous mind-wandering and obsessive compulsive symptomatology (see Figure 2 below).

## Discussion

The present study found that Covid-related stress partially mediated the relationship between spontaneous mind-wandering and obsessive-compulsive symptomatology, and that spontaneous mind-wander related to poorer psychological wellbeing. The finding that Covid-related stress partially mediated the relationship between spontaneous mind-wandering and obsessive-compulsive symptomatology was perhaps the most notable, as it provides information regarding the tangible effect Covid-19 has had on the mental health of the Irish population. Additionally, it highlights the role played by confounding variables in determining the relationship between MW variables and wellbeing outcomes. The mediating effect of Covid-related stress on the relationship between spontaneous MW and OCS is supported by research that firstly, links this form of MW with stress outside of the Covid context (Seli et al., 2019). Seli and colleagues propose a neurophysiological explanation for this association, in that chronic stress may influence attentional control (Liston et al., 2009), thus potentially resulting in increased experience of episodic unintentional MW. Conversely, it is also plausible that the boredom potentially elicited as a result of the behavioural restrictions associated with lockdown measures (e.g. Boylan et al., 2021) has contributed to an increase in the experience of spontaneous MW, and thus stress related to the present context. Our findings are also grounded in research that demonstrates the negative effect of both unintentional MW (Seli et al., 2017) and Covid-related stress (Cox & Olatunji, 2020) on OCS. The default mode network, and its association with both the experience of mind-wandering (Kucyi et al., 2013) and of OCD (Stern et al., 2012) may also provide an explanation for the observed relationship between both phenomena, and may prove an interesting avenue for exploration in future research. Notably, deliberate MW demonstrated no significant relationship with OCS; a finding that adds to the growing body of research that advocates for the separation of MW into its underlying subtypes as a more effective means by which to conceptualise the construct (Seli et al., 2017). Furthermore, our results highlight that while those reporting increased rates of spontaneous MW experience higher levels of OCS, the same does not apply to those who actively choose to mind-wander. As such, it is not merely the case that those reporting increased rates of OCS in the context of Covid-19 are more likely to mind-wander than those reporting lower levels of OCS, but rather that their experience of OCS relates to unintentional shifts in attention from the present environment to task-unrelated thoughts.

The finding that increased spontaneous MW related to lower subjective psychological wellbeing after controlling for Covid-related stress was consistent with previous research that has demonstrated the negative effect this form of MW has on wellbeing outcomes (e.g. Seli, et al., 2015a; Seli et al., 2017; Seli et al., 2019). Although the present study also explored the effect of unintentional MW on OCS within the Covid context, it appears to be the first to explore its association with subjective psychological wellbeing, suggesting that spontaneous MW not only effects psychopathology, but also overall psychological wellbeing. Of particular interest, our findings demonstrate that the relationship between spontaneous MW and psychological wellbeing remains consistent even after a third variable, i.e. Covid-related stress, is controlled for. Our findings provide evidence that spontaneous MW in itself may influence psychological wellbeing, as its negative association with wellbeing persists even after the effect of third variables that have demonstrated a significant relationship with wellbeing are removed. The contribution of Covid-related stress to poorer psychological wellbeing as identified in the present study demonstrates the influence contextual factors may have on the relationship between MW and wellbeing outcomes, and so determining the true nature of the relationship between the former and the latter variables may require researchers to account for additional factors that may be at play.

In the present study, although when included in a model with both other facets of MW daydreaming frequency negatively predicted subjective psychological wellbeing and increased the ability of the model as a whole to predict wellbeing, when taken alone the phenomenon did not uniquely contribute to wellbeing scores. This finding is in contrast with a wealth of previous research that has highlighted the many detrimental effects of increased engagement in MW on facets of psychological wellbeing (e.g. Killingsworth & Gilbert, 2010; Mar et al., 2012; Smallwood et al., 2007). It is possible that firstly, MW frequency alone cannot account for psychological wellbeing. Although, as previously outlined, earlier research has generally explored MW as a unitary construct encompassing frequency alone, more research is beginning to emerge separating it into its underlying subtypes. As such, it is conceivable that the combination of mind-wandering both frequently and spontaneously predicts lower wellbeing, while mind-wandering frequently alone, irrespective of the content or type, does not. This inconclusive result is, however, consistent with those of other studies that have struggled to find a significant or powerful effect of MW frequency on wellbeing outcomes (e.g. Johannes et al., 2018; Marchetti et al., 2012; Mason et al., 2013).

A similar result was observed for daydreaming about close family and friends, in that it neither significantly contributed to the model’s predictive power nor uniquely predicted variability among wellbeing scores. There are several possible explanations for this inconclusive result. Firstly, although daydreaming about close family and friends may relate to life satisfaction (Mar et al., 2012), it is possible that the same does not apply to psychological wellbeing. Life satisfaction and subjective psychological wellbeing are inextricably linked (Lombardo et al., 2018; Maddux, 2018), yet it must be acknowledged that they each represent distinct emotional and psychological experiences that are in turn preceded by different life events. Although there are myriad scales measuring wellbeing, it is generally defined by a more specific set of variables (e.g. negative and positive affect, engagement, relationships, meaning and purpose, achievement; Seligman, 2018) than is life satisfaction, which can encompass an infinite number of factors. It is therefore possible that although daydreaming content may influence life satisfaction, the same does not apply to psychological wellbeing. It is also possible that the Covid-19 pandemic has altered the relationship between MW content and wellbeing, in that before the pandemic daydreams centring around those whom we are close to may have induced positive emotions and increased life satisfaction. Conversely, during lockdown, spending an increased amount of time thinking about family and friends may exacerbate feelings of loneliness and isolation due to the uncertainty surrounding when seeing them again will be possible. Moreover, in the context of a pandemic, the content of daydreams related to close family and friends may have largely centred around fear of them becoming ill with Covid, the consequences of them becoming ill with Covid, or guilt about spreading it to loved ones, among other themes. There is some research to suggest that the affective valence of daydreams can influence emotional outcomes (e.g., Wen et al., 2022). Thus, it is important to acknowledge the effect of emotional valence on the content of daydreaming, as well as the resulting consequences, when considering explanations for the results of the present study. Exploring this possibility may require analysis of the interaction between Covid-related stress and daydreaming about close family and friends; thus providing an interesting avenue of research that may be worth exploring in the future. Finally, the relationship identified between daydreaming content and life satisfaction by Mar and colleagues was merely correlational in its nature, and therefore precludes the ability of researchers to make causal inferences. More complex analysis may therefore be required to determine the true nature of the relationship existing between MW content and wellbeing. However, it is worth noting that neither potential explanation need be classed as mutually exclusive, as MW may act as a both an antecedent to, and a consequence of poor wellbeing (Mason et al., 2013).

### Limitations

There are several limitations associated with the present study that necessitate addressing. Data collection took place while the population was enduring a third lockdown, during which universities, non-essential retail, and hospitality were closed down. The results obtained in the present study therefore represent the wellbeing of the sample while the pandemic was still ongoing. Although this provides valuable information regarding the in-the-moment psychological consequences of the Covid-19 pandemic, it is possible that research published in the coming year, now that measures have been lifted across the majority of the world since mid-2022, may indicate that these symptoms will somewhat subside. However, in a longitudinal study that compared symptoms among OCD patients at three distinct time points (i.e. baseline, early-Covid and one-year follow up), obsessive-compulsive symptoms that had worsened during the ‘early-Covid’ period persisted at the one-year follow-up (Liao et al., 2021). While the data collected at the one-year follow-up was done so while the pandemic was still ongoing, it provides tentative evidence to suggest that symptom severity may endure past the initial stages of development. Additionally, data was collected through online questionnaire, and participants were self-selecting. As such, the reliance of the present study on self-report, and thus its susceptibility to bias and fabrication, is important to acknowledge.

Despite the above mentioned limitations, the results of the present study have several strengths and practical implications. Perhaps most notably, it sheds light on the cognitive mechanisms underlying the relationship between spontaneous mind-wandering and obsessive-compulsive symptomatology, and the role played by stressors related to the Covid-19 pandemic in determining the strength of this relationship, thus extending the findings of previous studies that have explored these constructs separately. It highlights that it is not MW itself that predicts adverse psychological outcomes, but rather specific subtypes of the phenomenon that does so. Additionally, it provides a snapshot in time of how the wellbeing of the general population has been influenced by increased experience of stress as a result of Covid-19, and how this relates to mind-wandering. As noted, previous studies in the aftermath of the onset of the present pandemic have thus far failed to consider MW as a potential predictor of wellbeing.

### Recommendations and Future Directions

At present, we still have no information pertaining to how the Covid-19 pandemic has influenced rates of MW among the general population. Some work has been done exploring the buffering effects of mindfulness in the present context, in that engaging in the practice may protect against the harmful effects of Covid stressors that cause issues such as psychological distress, sleep disturbance and work engagement (Conversano et al., 2020; Zheng et al., 2020). Applying this information to the study of OCS that has been preceded by Covid may provide further insight into the most effective means by which to tackle this growing issue. Further research may also build upon the results of the present study by applying the methods to a different context or population sample. A considerable amount of research has begun to accumulate conveying the detrimental effects of Covid-19 on the psychological health of individuals with existing psychiatric diagnoses (Hao et al., 2020; Iasevoli et al., 2021; Muruganandam et al., 2020). Additionally, such individuals may be more susceptible to the implementation of maladaptive daydreaming as a means by which to cope during the pandemic (Musetti et al., 2021). As such, future research may apply the principles of the present study to a sample of individuals with an existing psychiatric diagnosis to determine whether the relationship between facets of MW and wellbeing outcomes differs among this population.

## Conclusion

To conclude, the present study has demonstrated a clear link between spontaneous mind-wandering and both subjective psychological wellbeing and obsessive-compulsive symptomatology in the context of the Covid-19 pandemic. Additionally, we have highlighted that it is not mind-wandering in itself that serves to explain poor wellbeing among non-clinical samples, but rather specific subtypes of the phenomenon that determine increased experience of obsessive-compulsive symptomatology and lower subjective psychological wellbeing. These findings represent an additional step towards obtaining a deeper understanding of the cognitive mechanisms underlying the psychological response of the general population to the COVID-19 pandemic, but a considerable amount of further research is required to ensure an overall more comprehensive overview is obtained.

## Data Availability

The datasets generated during and/or analyzed during the current study are available from the corresponding author on reasonable request. https://doi.org/10.6084/m9.figshare.24049662
